# The neural bases of vertebrate motor behaviour through the lens of evolution

**DOI:** 10.1098/rstb.2020.0521

**Published:** 2022-02-14

**Authors:** Shreyas M. Suryanarayana, Brita Robertson, Sten Grillner

**Affiliations:** ^1^ Department of Neuroscience, Karolinska institutet, 17177 Stockholm, Sweden; ^2^ Department of Neurobiology, Duke University Medical Center, Durham, NC 27710, USA

**Keywords:** lamprey, evolution, motor systems, networks, striatum, cortex

## Abstract

The primary driver of the evolution of the vertebrate nervous system has been the necessity to move, along with the requirement of controlling the plethora of motor behavioural repertoires seen among the vast and diverse vertebrate species. Understanding the neural basis of motor control through the perspective of evolution, mandates thorough examinations of the nervous systems of species in critical phylogenetic positions. We present here, a broad review of studies on the neural motor infrastructure of the lamprey, a basal and ancient vertebrate, which enjoys a unique phylogenetic position as being an extant representative of the earliest group of vertebrates. From the central pattern generators in the spinal cord to the microcircuits of the pallial cortex, work on the lamprey brain over the years, has provided detailed insights into the basic organization (a *bauplan*) of the ancestral vertebrate brain, and narrates a compelling account of common ancestry of fundamental aspects of the neural bases for motion control, maintained through half a billion years of vertebrate evolution.

This article is part of the theme issue ‘Systems neuroscience through the lens of evolutionary theory’.

## 1. Introduction

**Figure 1.**
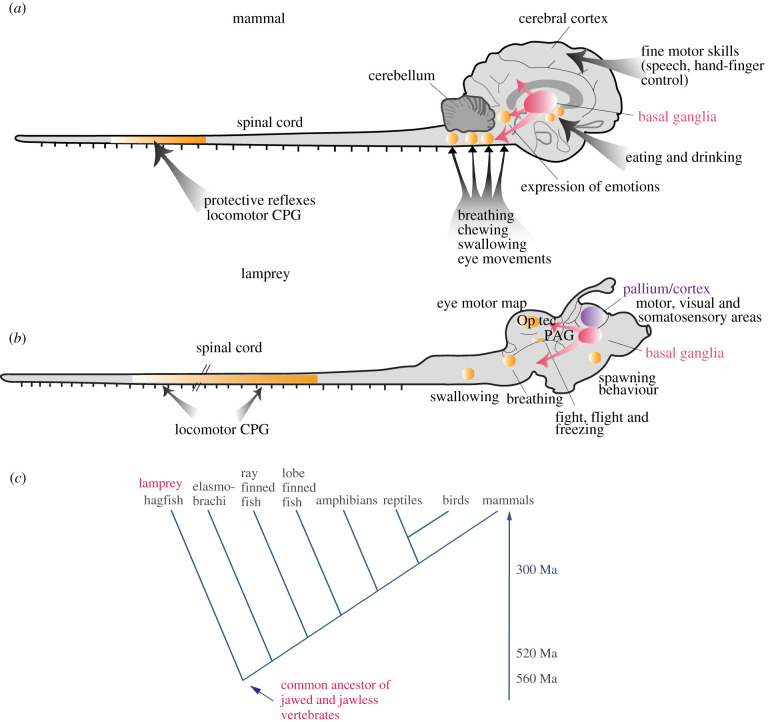
Common motor infrastructure from lamprey to man. (*a*,*b*) Throughout vertebrates, several basic motor behaviours are controlled by neuronal networks (central pattern generators, CPGs) located in the midbrain, brainstem (e.g. swallowing, breathing) and the spinal cord (e.g. locomotion). The pallium/cortex has evolved as the highest processing centre for multimodal sensorimotor control in the lamprey (*b*) and mammals (*a*) with the addition of fine motor control in the neocortex of primates (*a*). The basal ganglia are similarly organized in lamprey (*b*) and mammals (*a*) and play a crucial role in the selection of motor behaviours. (*c*) Phylogenetic tree showing that the lamprey diverged from the line leading to mammals around 560 Ma.

Among vertebrates, there is a gradual development of the movement repertoire from fishes and amphibians to that of birds and mammals. The lamprey, belonging to the oldest group of now living vertebrates, has only a limited set of motor behaviours such as locomotion, predation and feeding, while a cheetah, a ballet-dancer or a pianist have a varied and versatile movement repertoire. Below we will compare the nervous system of the lamprey with that of mammals, representing two phylogenetic extremes, to explore how much is common and what may have been modified from the point around 560 Ma, when the evolutionary line of lampreys became separate from that of mammals (figure 1*c*). We will report that the basic neural organization is remarkably similar between these two groups, although the number of neurons has increased markedly in practically all structures and so has the behavioural repertoire.

We will first consider the neuronal networks underlying the actual execution of movements located mostly in the spinal cord, brainstem or midbrain—and the basic neural organization, ‘the motor infrastructure’, in the lamprey and in mammals (figure 1*a,b*). In the second part, we will consider the forebrain (cortex, basal ganglia, thalamus, habenula) and its role in selection of action and decision-making and for recruiting the motor programmes needed at a particular point of time to meet intrinsic or environmental demands.

## 2. The motor infrastructure for execution of movement

The different neural networks underlying the execution of movement from the midbrain to the spinal cord in both the lamprey and mammals is shown in figure 1. The networks coordinating locomotion are located in the spinal cord and become activated from locomotor command centres in the mesencephalon and diencephalon. In the brainstem, we have the different motor programmes for swallowing and respiration, and for chewing in mammals. In the midbrain, there are command structures for escape and freezing responses (periaqueductal grey, PAG), while the superior colliculus (tectum in non-mammalian vertebrates) coordinates eye, orienting or evasive movements. These and other motor programmes serve as a motor infrastructure and can be recruited when needed. Each type of movement can be triggered and executed at this level, but the movements will not be adapted to the needs of the animal or be goal-oriented, which is a task for the forebrain. All vertebrates have a cerebellum although in the lamprey, cerebellum is rudimentary [1] with only a dorsal rim of tissue bridging the rostral part of the fourth ventricle, but it is already well developed in elasmobranchs.

### (a) The spinal cord and brainstem

If we consider the neuroanatomical underpinning, the spinal cord in mammals as well as the lamprey has sensory dorsal roots and ventral roots containing the axons of the motoneurons. The interneurons convey reflex action of the locomotor network in both groups. The lamprey has no appendages, and the body receives its innervation from the medial motor column like the trunk of mammals. Mammals also have a lateral motor column innervating the limbs [2]. The intrinsic function of the locomotor network in the lamprey, zebrafish and frog tadpole, which all move through undulatory swimming movements is understood, while the spinal networks for locomotion with limbs is not yet fully resolved [3–5]. In the lamprey, motoneurons and interneurons receive monosynaptic glutamatergic excitation from reticulospinal neurons in four different reticulospinal nuclei (the anterior rhombencephalic reticular nucleus (RRN) at the level of isthmus, middle RRN at level of nVII, posterior RRN at the level of nX and the mesencephalic reticular nucleus), which convey the excitation from the upstream locomotor command centres [6,7], as in mammals [8]. In mammals, the organization of the different reticulospinal nuclei is more elaborate considering the additional control of the limbs [9], and the transmission of excitation to the locomotor networks is mediated by one particular part of the reticulospinal system—the lateral gigantocellular nucleus, although other nuclei are also known to contribute [10,11]. In both species, somatosensory information is conveyed via the dorsal columns and the dorsal column nuclei to the contralateral thalamic nuclei, and further to cortex [12,13]. There is also a separate somatosensory system that has projections to the mesencephalic level in the lamprey [14], but whether they extend all the way to thalamus has not been shown, which is the case in mammals.

At the brainstem level, the different cranial nerves are organized in the same way in both groups, although for instance, the VIII nerve of the lamprey conveys not only vestibular information but also electroreceptive information from the surrounding space [15]. The eye motor nuclei (III, IV and VI) are organized similarly [16], as well as the trigeminal nerve (nerve V). The optic nerve (nerve II) projects to the thalamus and the superior colliculus and provides a retinotopic representation in both structures [13,17,18]. The olfactory input (nerve I) is organized with olfactory receptor cells projecting to separate glomeruli [19,20], and the information is carried further via two separate avenues—the mitral and the tufted olfactory projection neurons in both groups [21] (figure 3).

### (b) The respiratory networks controlling gills and lungs

Respiration is crucial for survival. In the lamprey, ventilation is produced by letting the water pass back and forth over gill pouches, while mammals have evolved lungs that requires air to move in and out. The requirement is to remove CO_2_ from the blood and add O_2_. In both cases, expiratory and inspiratory movements need to be generated. The lamprey paratrigeminal respiratory group is responsible for driving the respiratory movements [22–24], which in mammals is mediated by the preBötzinger complex [25]. An inactivation of the respective nuclei stops the respiratory movements in both mammals and the lamprey. Moreover, the two respiratory structures are very similar with an excitatory interneuronal network that generates the basic rhythm and is modulated by inhibitory neurons, while cholinergic and substance P neurons are also engaged in both structures. It thus seems that during evolution, the same central neural circuit has been kept to generate the respiratory rhythm despite the great changes that have taken place in the periphery [22–24].

### (c) Chewing, a mammalian speciality

Mammals have central pattern generator networks (CPG) in the brainstem that can be activated and modulated from the cortex, which include the networks controlling chewing. It appears that chewing is mainly a mammalian capacity, while other animals mainly bite and swallow as for instance, the crocodile. All vertebrates have the capacity to swallow, and there is a brainstem swallowing network that moves the food from the mouth to the ventricle or similar structures. In mammals, the different oral and pharyngeal muscles are activated in a precise sequence owing to a swallowing CPG network [26] (for review, see [27]). In the lamprey, the feeding behaviour has been studied in terms of sucking movements combined with swallowing, but no analysis of the underlying neural circuits has been performed [28,29]. The general motor pattern of swallowing may be similar in all vertebrates although the organization of peripheral organs in terms of e.g. the ventricle or craw, differ.

### (d) Tectum/superior colliculus directs movements to different points in the surrounding space

Moving to the midbrain, the optic nerve terminates as a retinotopic map in the superficial layer of the tectum/superior colliculus, providing information of the surrounding space, with the recipient neurons responding to salient stimuli in a particular location [15,17]. In a somewhat deeper layer, other senses provide maps aligned spatially with the visual map, such as electrosensation in the lamprey or auditory stimuli in mammals. Input originating from the same location in space provided from two different senses facilitate each other, and if instead they are in conflict, they inhibit each other both in the lamprey and in rodents [15,30–32]. The aligned spatial information from a given point in space will directly or indirectly excite the tectal output neurons, while at the same time they inhibit neurons in the surrounding tectal area with a form of lateral inhibition.

In congruence with the sensory map, there is a motor map able to evoke saccadic eye movements to a specific location in the environment. The motor map can direct the eyes to any point of the surrounding visual space and also elicit orienting movements of the head and body. A separate set of output neurons from the tectum elicit avoidance movements that lead to a movement in the opposite direction, as to avoid a collision. This is equally important behaviourally and required when walking along the pavement and one needs to avoid bumping into fellow pedestrians or if as a bird, flying through foliage. Looming stimuli that increase in size are perceived as threatening and very effectively activate the same evasive output neurons [33,34]. The looming stimuli are processed in the tectum and if it is inactivated, the looming response is abolished. Salient stimuli recorded in the tectum are efficient in activating dopaminergic neurons in the substantia nigra pars compacta (SNc) in both groups [35–37]. The information processed in the tectum is also projected further to the thalamus both in the lamprey and mammals and at least in the latter group also onwards from the thalamus to cortex. In addition, the thalamus projects back to the tectum (superior colliculus) [13,35].

### (e) Periaqueductal grey and griseum centrale: a command centre for defence reactions

PAG is composed of the grey matter surrounding the canal between the third and the fourth ventricle in mammals. It is called griseum centrale in the lamprey and zebrafish. Stimulating different parts of PAG in a variety of mammals can elicit escape responses, freezing and threatening vocalizations [38] and suppression of pain. In the zebrafish, griseum centrale is associated with defence reactions and freezing [39]. The connectome of griseum centrale is practically identical in the lamprey and zebrafish with outputs to the SNc and the pretectum and inputs from the SNc, pallium/cortex, hypothalamus and from 5-HT neurons in the raphe. The lamprey *griseum centrale* is also virtually identical to that of the mammalian PAG, which in addition receives input from the amygdala, a fear-related structure not yet identified in the lamprey, although homologous areas have been suggested in the zebrafish [40,41].

### (f) Dopamine innervation: substantia nigra pars compacta

**Figure 2.**
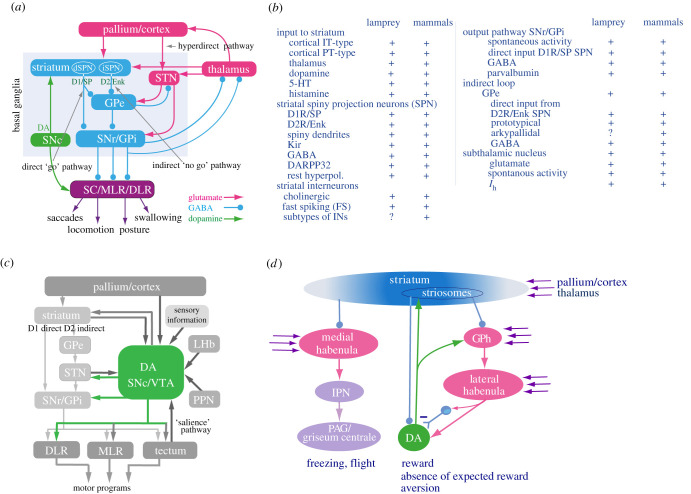
The lamprey basal ganglia. (*a*) The organization of the basal ganglia. The striatum consists of GABAergic neurons, as do globus pallidus pars externa (GPe), globus pallidus pars interna (GPi) and substantia nigra pars reticulata (SNr). SNr and GPi represent the output level of the basal ganglia, and they project via different subpopulations of neurons to the tectum/superior colliculus, the mesencephalic (MLR), and diencephalic (DLR) locomotor regions and other brainstem motor centres, as well as back to thalamus with efference copies of information sent to the brainstem. The direct striatal projection neurons (dSPNs) that target SNr/GPi express the dopamine D1 receptor (D1) and substance P (SP), while the iSPNs (indirect striatal projection neurons) express the dopamine D2 receptor (D2) and enkephalin (Enk). Also indicated is the dopamine input from the SNc (green) to striatum and brainstem centres. Excitatory glutamatergic neurons are shown in pink and GABAergic structures in blue. (*b*) A table showing the key features of the basal ganglia organization that are found in mammals and the lamprey. (*c*) The substantia nigra pars compacta (SNc) connectome in the lamprey and mammals. The efferent and afferent connectivity of the SNc is virtually identical. (*d*) The striatum targets both the medial and lateral habenulae. The medial habenula sends projections to the interpeduncular nucleus (IPN) and further to the PAG/griseum centrale. In mammals, PAG triggers a variety of fixed action patterns related to freezing and flight. The lateral habenula is engaged in the control of the different modulator systems and receives input from the glutamatergic part of globus pallidus (GPh), which in turn receives inhibition from the striosomal compartment of the striatum. The striosomes, GPh and the lateral habenula itself receive input from the pallium, thalamus and other structures.

In mammals, the SNc provides the dopamine innervation to the lateral parts of the dorsal striatum, while the ventral tegmental area (VTA) innervates the rostral parts of the dorsal striatum and the ventral striatum. SNc and VTA are located close to each other in the mesodiencephalon, while the SNc in lamprey is located in the most caudal part of the diencephalon. The dopamine neurons in the lamprey SNc most likely share their developmental origin with the mesodiencephalic dopamine neurons of the SNc/VTA in amniotes [42,43]. The connectome of SNc, shown in figure 2*c*, is practically identical in lamprey and mammals [36,44–46]. Thus, the SNc innervates striatum and other parts of the basal ganglia, and the downstream motor targets such as the superior colliculus/tectum and the locomotor command centres in the mesencephalon in both groups. The SNc gets input from the striosomes in striatum, the lateral habenula, pedunculopontine nucleus, tectum and pallium/cortex. SNc neurons respond with a burst of activity to salient stimuli in the environment mediated via the tectum/superior colliculus in the lamprey and mammals [35–37]. An individual dopamine neuron that innervates striatum also sends a branch to downstream motor centres and potentiates visuomotor responses and the initiation of locomotion. An SNc burst will thus potentiate the downstream motor centres before the action of the striatum has had the time to become manifested [35–37,47,48].

In conclusion, the neuronal networks and general motor infrastructure underlying the execution of the basic motor repertoire in the spinal cord, hindbrain and midbrain is organized in a similar way in mammals and the lamprey - the *‘bauplan’* is virtually identical.

## 3. Forebrain control: selection of behaviour, decision-making and learning

### (a) Thalamus: a sensory communication hub

The thalamus is a critical communication hub in the forebrain, being a major source of information to the cortex and striatum from subcortical and peripheral sources. Without the thalamus, the cortex is blind, deaf, cannot perceive touch and is essentially detached from the sensory world. All sensory information except for olfaction is channelled via the thalamus to the cortex. The organization of thalamus in mammals is in the form of nuclei; there are over 30 distinct nuclei in the mouse thalamus that target different cortical or striatal areas. The thalamus develops from prosomere p2, as is also the case in the lamprey [49]. The thalamopallial neurons are glutamatergic and excitatory [50] and located in both the periventricular and lateral parts, and are interspersed with GABAergic neurons. In the mouse, there are a few thalamic nuclei which have a small proportion of interspersed GABAergic neurons, but the major source of GABAergic input is from the reticular nucleus [51]. In the lamprey, the location of the thalamostriatal neurons is in the periventricular area, whereas the thalamopallial neurons are located both in the periventricular and lateral areas. The thalamus does not form distinct nuclei in the lamprey and this scenario echoes through several other anamniote classes where distinct thalamic nuclei are difficult to discern, as there are relatively fewer cells.

### (b) Thalamic pathways through vertebrate evolution

The sensory pathways through the thalamus can be more readily compared across vertebrates [52]. A major sensory pathway is the visual relay. In the lamprey, thalamocortical cells receive retinal input onto their dendrites which extend into the optic tract where they form synapses with retinal afferents [13]. There is also a retinotopic organization in the optic tract, with retinal afferents targeting both the thalamus and the rostral and caudal optic tectum [17]. This retinotopic specificity is also represented in the thalamus, which is in turn relayed to and maintained in the visual pallium [13]. This pathway is comparable to the mammalian primary visual pathway that relays retinal information via the dorsal lateral geniculate nucleus to the primary visual cortex. It is however, not yet clear whether the different subtypes of retinal ganglion cells present in mouse are present in the lamprey. The role of the retino-thalamocortical pathway in lamprey is to relay the topographic representation of the visual space to the first-order thalamorecipient neurons in the visual cortex as in mammals. This pathway has progressively evolved to relay high-dimensional data including high acuity vision in primates.

In addition to the retinothalamic pathway, there are also projections to the thalamus from the roof of the midbrain, in particular the optic tectum (superior colliculus) and pretectum [53,54] that target periventricular nuclei in mammals. These projections relay processed information from the tectum to cortex [15,53,55,56]. The retinothalamic and tectothalamic pathways target distinct cortical/pallial areas in mammals, reptiles and birds [52,56,57]. The tectal output neurons projecting to reticulospinal neurons in the lamprey, involved in the control of eye, orienting or evasive movements, also send projections to the thalamus [31,54,58]. These tectothalamic projections are important for visuomotor coordination, including mediation of visuomotor processing [15]. Neurons in the pretectum, the orchestrators of optokinetic reflexes, also send collaterals to the thalamus [54]. It is however, not known whether the information from these two pathways in the lamprey are mediated by distinct subpopulations of thalamopallial neurons as in amniotes, although this seems likely.

In addition to the visual pathways, somatosensory information is mediated via the trigeminal sensory nucleus and the dorsal column nuclei, respectively, to separate populations of neurons within the thalamus. They in turn project to separate adjacent parts of the somatosensory dorsal pallium to provide a somatotopic representation [13]. The equivalent pathway in mammals is via the ventroposterior nucleus, a major relay of somatosensory information to the primary somatosensory cortex, which has been identified in all examined mammals [59]. Similar pathways are known to exist in reptiles and birds [60]. The lamprey data and available data from other vertebrate clades highlight the possibility that the primary visual and somatosensory pathways are consistent across vertebrates from the lamprey and elasmobranchs to basal teleosts, amphibians and amniotes [52,57]. Modern teleosts have additionally evolved a novel sensory relay, the preglomerular nuclei, which may be of a different developmental origin and forwards information from the tectum to pallium [61].

Other than with the pallium/cortex, a major interaction of the thalamus is with the basal ganglia. This includes projections of the thalamus to the striatum, as well as the input to thalamus from the substantia nigra pars reticulata (SNr). The SNr also projects to different midbrain and brainstem motor centres and sends axonal collaterals to the thalamus [62]. The thalamostriatal projections have been identified in most vertebrates including the lamprey [63]. In mammals, the parafascicular and central lateral nuclei, which receive input from the superior colliculus, pedunculopontine and the basal ganglia output nuclei, project to the striatum [52,57,64]. In birds and non-avian reptiles, the thalamostriatal pathways arise from the nucleus rotundus, the medial complex and the posterior dorsolateral nucleus [56,60].

**Figure 3.**
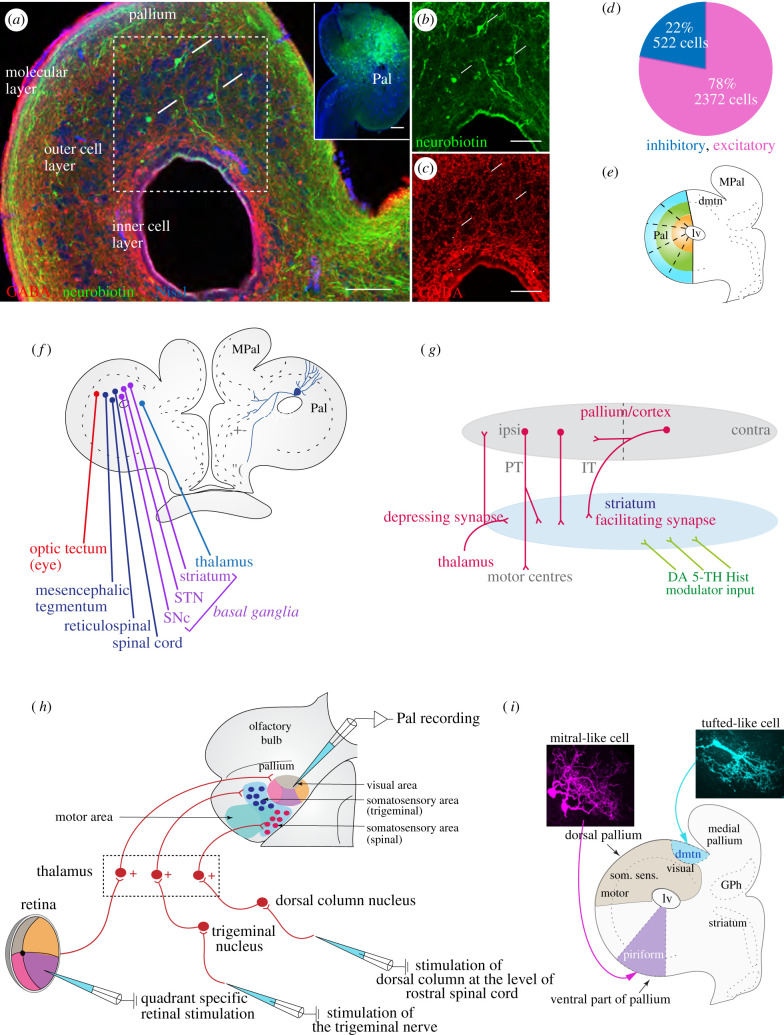
Cell types in the lamprey pallium/cortex. (*a*) Retrogradely labelled intratelencephalic type (IT-type) cells (arrows) following neurobiotin injection in the contralateral pallium (inset). (*b* and *c*) The IT-type cells (*b*, arrows), from the region of dotted square in (*a*) do not express GABA (*c*, arrowheads). Note the three different layers, the molecular layer, and the outer and inner cellular layers. (*d*) Overall percentage and number of GABAergic and non-GABAergic pallial neurons. (*e*) Schematic of the lamprey pallium with segments, indicated with dotted lines, used for cell counting (Pal, pallium; MPal, medial pallium; lv, lateral ventricle). (*f*) Schematic of a transverse section of the lamprey pallium indicating the efferent targets of pallial projection neurons (PT-type, pyramidal tract type). (*g*) Projections of PT- and IT-type pallial neurons in the lamprey. (*h*) Summarizing schematic of the lamprey dorsal pallium, showing retinotopic visual areas, somatosensory areas and motor areas, as well as the retinal, trigeminal and dorsal column nucleus afferents relayed via distinct subpopulations of thalamic neurons. (*i*) The olfactory system in lamprey resembles that of mammals in many respects with a dual efferent system from the main olfactory bulb conveyed via tufted- and mitral-like cells. The latter target the olfactory cortex located in the ventral pallium, whereas the former target a separate limited region, the dorsomedial telencephalic nucleus (dmtn). Note the different morphology of the mitral-like (magenta) and tufted-like (blue) cells.

Cortical modulation of thalamic activity constitutes a major aspect of the thalamocortical projectome. The ‘first-order’ relay neurons or sensory thalamic neurons receive massive afferent input from peripheral and subcortical structures as ‘driver input’, and ‘modulator input’ from layer 6 neurons of the neocortex. By contrast, ‘higher-order’ relay neurons receive ‘driver input’ from layer 5 cortical neurons, as an efference copy of their output to subcortical motor centres in the brainstem and spinal cord [65,66]. The lamprey dorsal pallium has projection neurons (pyramidal tract/PT-type), which target all downstream motor centres including the thalamus and the brainstem [67] (figure 3*f*). However, whether there are exclusive projections to the thalamus from the pallium is not known. In both reptiles and birds, pallial projections are present to the thalamus from the dorsal cortex and Wulst/dorsal ventricular ridge, respectively [60,68,69].

In conclusion, the thalamus has evolved to serve as an essential and critical interactive hub in the forebrain in vertebrates extending from the lamprey to primates. It forwards primary sensory and also processed information in parallel to cortex and striatum.

### (c) Basal ganglia: action selection and initiation

A group of subcortical nuclei in the forebrain, the basal ganglia (figure 2*a*, which applies to both lamprey and mammals), play vital roles in selection of action, motor learning and decision making. Recent data from the lamprey, which has detailed the presence of the basal ganglia in terms of subnuclei, connectivity, cell types and neurotransmitters, now allow comparisons regarding the evolutionary trajectory of the basal ganglia through vertebrate evolution (figure 2*a*,*b*). The basal ganglia have been shown to be evolutionarily conserved beginning with the earliest group of vertebrates [63,70].

#### (i) Striatum: cell types and connectivity

The main input structure of the basal ganglia in mammals and lamprey is the striatum (figure 2*a*), and it is constituted largely of GABAergic spiny projection neurons (SPNs), which can be divided into the dopamine D1/substance P or D2/enkephalin receptor-expressing subtypes [71]. The D1 SPNs project to the output nuclei, the globus pallidus pars interna (GPi) and SNr, forming the ‘direct pathway’, whereas the D2 SPNs project to the globus pallidus pars externa (GPe) and the subthalamic nucleus (STN), which in turn project to the output nuclei [63,64]. The net effect of the direct pathway is to promote movement, whereas the indirect and hyperdirect (see below) pathways counteract motion. The list in figure 2*b* shows the detailed similarities in general organization of all parts of the basal ganglia.

Striatal SPNs usually rest at hyperpolarized membrane potentials owing to inward rectifying potassium channels and receive their dopamine inputs from the SNc with the differential effects induced by the D1 and D2 subtypes [44,72]. The organization of the striatum into striosomes and matrix can also be seen in the lamprey with the matrix expressing calbindin as a marker, similar to mammals [73]. The striatum receives inputs from both the pallium/cortex and thalamus, although the relative contributions of either to striatal function is not yet fully understood [13,67,73]. The input to striatum flows through distinct channels from across different regions of the neocortex, as well as via the parafascicular nucleus of thalamus to specific modules within striatum [74]. These different channels converge onto the output nuclei where there are much smaller number of neurons. The projections from the pallium/cortex to the brainstem (PT-type) target the striatum en route to their terminations in the brainstem, while the intratelencephalic (IT-type) projections, similar to mammalian callosal projections, target the contralateral pallium and striatum [50,67]. The periventricular thalamus provides glutamatergic input to the striatum in lamprey [13,73] and forms depressing synapses on SPNs as the parafascicular nucleus in rodents, in contrast to the pallial input, which forms facilitatory synapses [75,76].

Other than the SPNs, the striatum consists of a small but significant percentage of interneurons (5%) out of which seven types have been identified in mice based on recent RNA sequencing data [77]. The major classes are the cholinergic, fast-spiking and low-threshold spiking interneurons and there are other smaller subpopulations [78]. In the lamprey, cholinergic and fast-spiking interneurons have so far been identified [72,79,80]. The lamprey striatum also receives neuromodulatory inputs other than dopamine, including 5-HT and histamine as in mammals [64,70].

The overall evidence indicates that the striatum evolved as an important player in the selection and initiation of action through conserved circuit mechanisms mediated via the direct and indirect pathways. In the lamprey striatum, no strict subdivision can be made between the dorsal and ventral striatum, but it may be more akin to the dorsal striatum of mammals.

#### (ii) Globus pallidus pars externa and Subthalamic nucleus

The GPe and STN are two important nuclei of the basal ganglia, mediating the indirect pathway and their basic connectivity is present in both the lamprey and mammals (figure 2*a*,*b*). The GPe was long considered as a relay nucleus, but recent evidence suggests that it has a more active role in basal ganglia function [81–86]. The STN is glutamatergic and in addition to being part of the indirect pathway, it receives input from the pallium/cortex and projects to SNr forming the ‘hyperdirect pathway’, which can stop ongoing actions [87]. Both the prototypical GPe neurons and STN neurons are spontaneously active at rest.

In rodents, a new subtype of GPe neurons, the arkypallidal cells have been identified, that project exclusively to the striatum providing a massive innervation [88]. Their activation can make a mouse stop to locomote and they are therefore called ‘stop cells’ [84,86]. Whether these cells are present in the lamprey is not known. The GPe is intermingled with the GPi in the lamprey as in birds, and the neurons are similar to the prototypical neurons of mammals [63,70,73].

#### (iii) Output nuclei: globus pallidus pars interna/substantia nigra pars reticulata

In both the lamprey and mammals, the output nuclei of the basal ganglia GPi/SNr are tonically active at rest and maintain a tonic inhibition of their downstream targets in the brainstem and the thalamus (figure 2*b*) [62,73,89]. The initiation of an action is through inhibition of a specific set of SNr neurons via the direct pathway, resulting in consequent disinhibition of the specific motor centre that they target [62,74,90,91]. Each SNr neuron has an axonal branch directed to thalamus that conveys an efference copy reflecting the signal conveyed to midbrain–brainstem motor centres. The thalamic activity relayed back to cortex and striatum is thus proportional to the downstream control signal. In rodents, the GPi is very small as compared with SNr. Inputs from the striatal direct and indirect channels converge onto channels formed by subsets of GPi/SNr neurons [74].

In the lamprey, the basal ganglia can be considered to have a set of modules that each control basic aspects of behaviour, such as locomotion, posture, orienting and eye movements [70]. Each module would have subsets of striatal and GPe neurons and a specific output level from GPi/SNr. During evolution, this basic control strategy has been retained (figure 2*b*), but with an increasing number of modules as the behavioural repertoire has gradually increased to the versatile primate behavioural repertoire. The larger number of modules, the more versatile the behavioural repertoire.

The modus operandi of the basal ganglia—selection/initiation of action through disinhibition of downstream targets is an evolutionarily well-conserved mechanism in motor control, which evolved very early in vertebrate evolution [70]. It would seem that the strategy of tonic inhibitory control in the default state and allowing appropriate action when necessary through disinhibition, allows for better goal-directed behaviour by preventing unwanted motor actions from being accidentally activated.

### (d) The lateral and medial habenulae: action evaluation and flight/fight behaviours

A critical aspect of the neural basis for action is the evaluation of the outcome of an action. The basal ganglia and the habenulae in interaction with the dopamine system play critical roles in this respect. The habenulae comprised a medial and a lateral part, with a connectivity that is conserved across vertebrates [92,93]. The right part of the diagram in figure 2*d* shows that striatal projection neurons in the striosomal compartment project directly to the dopamine neurons and can therefore suppress their activity. There are also other striosomal neurons that project to the excitatory subset of neurons in the globus pallidus that in turn project to the lateral habenula (GPh—globus pallidus projecting to the habenula [73]). The lateral habenula projects directly to the dopamine neurons but in parallel with an inhibitory link that appears to dominate. The GPh can be inhibited from the striosomes and excited by the pallium and thalamus in both the lamprey and mammals [73,94]. The net effect is that an increased activity in GPh will excite the lateral habenula, which will mainly inhibit the SNc. The converse will happen with a decreased activity in GPh that will result in an enhanced activity in the dopamine neurons (reward-like). Different subpopulations of cells in the lateral habenula control the level of activity in the dopamine neurons in SNc, and the 5-HT and histamine systems [93].

The medial habenula receives different types of sensory inputs in the lamprey ranging from olfactory, pineal, vestibular and electrosensory inputs. In both the lamprey and zebrafish, inputs from the medial habenula target the interpeduncular nucleus (IPN), which in turn projects to the griseum centrale [39,40]. The medial habenula also targets the IPN in mammals and plays a role in mediation of fear-related behaviour [38] (see above for PAG/griseum centrale).

The organization of action evaluation circuits and fear responses including flight/fight behaviours are thus part of an ancestral circuit design mediated by the two habenulae acting through their selective control of the different modulatory streams of dopamine, 5-HT and histamine neurons, respectively, each supplying inputs to the striatum.

### (e) Pallium/cortex: sensorimotor planning and perception

Evolutionary ancestry of a brain structure can provide critical insight into functional constraints shaping its cytoarchitecture, connectivity and microcircuit features. The pallium/cortex in mammals consists of laminated structures, some of which are three-layered such as the piriform cortex and hippocampus and the more elaborated six-layered neocortex. The evolutionary ancestry of the neocortex, the most recent and complex product of evolution of the nervous system, has been a matter of debate for over a century. Until recently, the common wisdom was that the mammalian neocortex originated from its common reptilian ancestors over 300 Ma. Modern reptiles have a three-layered dorsal cortex, which was considered homologous and ancestral to the mammalian neocortex [95]. The pallia (cortices) of anamniotes (cyclostomes, fishes, amphibians—so-called ‘lower’ vertebrates) were considered to be mainly or entirely olfactory, without any structure homologous to the mammalian neocortex [96,97]. However, recent studies have revealed that the evolutionary ancestry of the neocortex can be stretched back to the dawn of vertebrate evolution, in terms of basic connectivity, sensorimotor topography and function.

#### (i) Cytoarchitecture, connectivity and cell types

The lamprey pallium/cortex is three-layered (figure 3*a*), with an outer molecular layer largely devoid of neurons, apropos to the molecular layer (layer 1) of the mammalian cortex, and a cellular layer, which can be subdivided into two parts based on the relative distribution of GABAergic neurons and general cell density. The ‘inner cellular layer’ consists of a higher proportion of GABAergic cells and an ‘outer cellular layer’ with a smaller proportion of GABAergic cells but a higher cell density than the inner cell layer. The overall proportion of GABAergic neurons in pallium is about 22%, which is roughly the same proportion as in mammals (figure 3*d*,*e*). Subtypes of GABAergic interneurons, calbindin- and calretinin-expressing interneurons are present [50]. The neocortical homologue in non-avian reptiles, the dorsal cortex, and the olfactory lateral cortex are also three-layered [50]. While the presence of pallial cortices in other anamniotes remains to be ascertained, the three-layered dorsal pallium in the lamprey suggests the possibility of the six-layered neocortex having evolved from the elaboration of an ancestral three-layered cortex, with the addition of layer 2/3. In mammals, the number of cortical columns has increased dramatically from mice to humans, with a corresponding increase in cognitive ability. This is also accompanied with the expansion of layers 2/3 in primates.

Another useful way to look at the evolution of the neocortex is via the evolution of cell types. The major output cells of the neocortex, the layer 5b (PT-type) cells, that target the brainstem and spinal cord seem to have been conserved in terms of their projection pattern and function. They are the cells relaying the cortical command to downstream motor centres and crystallize the role of neocortex in terms of a ‘broadcaster of motor commands' [98–100]. These projections arise from sensory and motor areas of the neocortex and they also do so in the dorsal pallial homologues in non-mammalian amniotes, including the dorsal cortex of reptiles and the avian Wulst [101] as well as in some anamniote species like sharks [102]. These PT-type projections are also present in lamprey and target all major downstream motor centres (figure 3*f*); and indeed, electrical stimulation of the motor area of the dorsal pallium in lamprey gives rise to well-delineated movements of the eyes, mouth, trunk and locomotion [67], as in mammals. In addition, the glutamatergic IT-type cells also are conserved in terms of their projection pattern with their targets to the contralateral pallium and striatum in amniotes and lamprey (figure 3*a–c*,*g*). Another cell type which can be identified are the monosynaptic thalamorecipient neurons or the mammalian layer 4-equivalent cells, which have been found in the dorsal cortex of reptiles, in the avian Wulst, as well as in the lamprey [50,103,104]. Thus, broadly, the three basic cell types—output, input and IT cells are found in proposed dorsal pallial homologues in the lamprey and they are located in the cellular layer and have spiny dendrites extending to the molecular layer [50,103–105]. One could thus propose that the common amniote ancestor probably possessed a three-layered cortex with these three cell types as a conserved feature. Indeed, the results in the lamprey showing a three-layered cortex similar to that of the reptilian dorsal cortex supports this view. It would appear that evolution has retained the basic connectivity of these cell types and there has been a conspicuous increase in the number of these cells and a dramatic generation of new excitatory cell types in mammals in terms of their molecular identity. It is conceivable that these new molecularly defined excitatory cell types have emerged from ancestral ones, revealing a fundamental principle of the evolution of the neocortex [106]. Another aspect to consider here are the two distinct modes of cortical neurogenesis in mammals—direct and indirect, with indirect neurogenesis contributing to the expansion of the neocortex, particularly giving rise to number of neurons in the upper layers [107]. Indirect neurogenesis also occurs in non-mammalian amniotes like birds, but to a much lesser degree [108]. In the lamprey and other anamniotes, the mechanisms of pallial neurogenesis are unclear, but could be through the mode of direct neurogenesis.

#### (ii) Visual, somatosensory and motor representations in the pallium/cortex

The pallium of the lamprey was until recently assumed to be mainly olfactory, but as is clear from above, there is a distinct motor area with projections to the same downstream targets as in mammals (figure 3*f*,*h*) [67]. To our surprise, we could also show that there is a distinct visual area with a clear retinotopic representation (figure 3*h*,*i*) [13], similar to the mammalian visual area (V1). Moreover, there is a somatosensory area intercalated between the visual and the motor areas (figure 3*h*, blue and red dots) with a basic somatotopy, with distinct representations of the cutaneous inputs from the trigeminal (head) and dorsal column (body), respectively. Both the visual and somatosensory inputs are relayed via the thalamus with maintained topographic specificity [13]. These areas are located in the dorsal part of the pallium/cortex and are considered as the lamprey dorsal pallium (figure 3*i*) [13,109]. Distinct topographic visual, somatosensory and motor areas are a quintessential aspect of neocortical organization and has been demonstrated ubiquitously in all mammals examined [110]. Distinct representations of vision and somatosensation are also present in the dorsal cortex of reptiles and in the avian Wulst [95].

Other than sensorimotor topography and processing, an essential element to the function of the dorsal pallium is association. In the mammalian neocortex, there are higher-order areas or association areas which essentially process multimodal inputs. Whether specific association areas exist in the lamprey is unclear, but the output neurons of the dorsal pallium, the PT-type neurons, can integrate different modalities including information relayed from the olfactory bulb and the thalamus [50]. The dorsal pallium additionally receives processed olfactory information relayed from the olfactory areas in the ventral pallium (see below, [21]).

#### (iii) Olfactory representation in pallium/cortex

Olfactory stimuli are represented in the medial area of the ventral part of pallium (figure 3*i*) [21,111]. In the lamprey, it receives inputs from the mitral-like cells via the lateral olfactory tract, with short latency—a putative olfactory cortex, as in mammals. The lamprey tufted-like cells on the other hand, project separately and exclusively to a relay nucleus called the dorsomedial telencephalic nucleus (dmtn), which in turn, projects to a circumscribed area in the anteromedial olfactory cortex. In mammals, the tufted cells project to a small, circumscribed area near the olfactory tuberculum. Thus, the olfactory area in the lamprey pallium constitutes a ventral pallium (figure 3*i*) [21,109]. The lamprey ventral pallium is also three-layered and similar to that of mammals and has neurons with layer-spanning spiny dendrites [21,50].

#### (iv) Pallial sectors

The developing pallium can be divided into four distinct sectors, the dorsal, lateral, ventral and medial pallium [109] with the dorsal pallium forming the neocortex, the medial pallium the hippocampus, the lateral pallium forming the claustroinsular complex and the ventral pallium forming the olfactory centres. These four sectors have been identified across amniotes, while data in anamniotes has remained less clear. It is conceivable that the ancestry of at least two pallial sectors—the dorsal and ventral pallium could be described as pan-vertebrate based on our data from the lamprey [13,21,50,67,105]. It would appear that olfactory processing centres in pallium co-evolved with other sensory modalities such as vision and somatosensation. The dorsal pallium has evolved to be the highest centre of multimodal sensory processing and sensorimotor integration with the ability to target and control all major downstream motor control structures in mammals and seemingly across vertebrates.

## 4. Concluding remarks

Our conclusion from the results above is that the basic design of the vertebrate nervous system from the pallial cortex to the spinal cord had already evolved when the lamprey diverged from the evolutionary line leading to mammals. This applies to the very detailed similarity of the basal ganglia regarding connectivity transmitters/peptides and general organization (figure 2*a*,*b*). Similarly, the small lamprey pallium has a motor area with the same downstream projection pattern as in mammals, and a somatosensory and a retinotopic visual area. The midbrain is very similar with the tectum/superior colliculus organization for detecting salient stimuli from the surrounding space and being able to direct eye and orienting movements to a specific area. Furthermore, the midbrain–brainstem–spinal cord is central for the control of locomotion. This shows that the design of the nervous system has remained conservative, although the number of neurons and microcircuits have increased dramatically from lamprey to primates. The same architectural plan for the nervous system has been retained as the dimension of the brain has increased, or in other terms, the ‘*bauplan*’ has been kept the same.

Of course, major additions have occurred during vertebrate evolution with the limbs and the lateral motor column that is used for propulsion in tetrapods, as well as reaching and grasping, which also means that the neural circuits have had to further adapt the existing neural machinery to new challenges. Another example, basic neural circuits controlling respiratory movements appear identical although at the periphery, but there is a major transition from gill pouches to lungs. In addition, a concomitant evolution of new cell types has occurred through a diversification of ancestral ones, as well as microcircuits, to cater for species-specific behavioural requirements.

Thus, a rather complete *bauplan* had evolved with cyclostomes very early in vertebrate evolution. A major development occurred between the preceding stages with the amphioxus that has a brain vesicle with similar transcriptional factors as in the vertebrate forebrain, but only with few neurons and a limited forebrain organization. Recent data on the amphioxus indicates the presence of a possible telencephalic domain, the pars anterodorsalis, with glutamatergic and GABAergic neurons as well as dopaminergic neurons, which can be associated with forebrain structures of vertebrates such as the pallium, olfactory bulbs and the hypothalamus [112,113] as discussed by Lacalli [114]. It remains to be ascertained if this presumptive telencephalic domain, in the dorsal locus of the hypothalamus alar plate, is equivalent to the corresponding vertebrate prosomere [113,115]. There is, however, no evidence of major forebrain structures, like for instance the thalamus, and midbrain structures present in vertebrates. There is thus an impressive and enigmatic evolution that took place at the stages between the amphioxus and cyclostomes with an almost complete blueprint of the vertebrate motor system.

## Data accessibility

This article has no additional data.
